# Oxfordshire Women and Their Children's Health (OxWATCH): protocol for a prospective cohort feasibility study

**DOI:** 10.1136/bmjopen-2015-009282

**Published:** 2015-11-09

**Authors:** S Harrison, G Petrovic, A Chevassut, L Brook, N Higgins, Y Kenworthy, M Selwood, T Snelgar, L Arnold, H Boardman, C Heneghan, P Leeson, C Redman, I Granne

**Affiliations:** 1 Nuffield Department of Primary Care Health Sciences, University of Oxford, Oxford, UK; 2 Nuffield Department of Obstetrics & Gynaecology, University of Oxford, Oxford, UK; 3 Cardiovascular Clinical Research Facility, University of Oxford, Oxford, UK

**Keywords:** pregnancy, pre-pregnancy, maternal health

## Abstract

**Introduction:**

Some specific pregnancy disorders are known to be associated with increased incidence of long-term maternal ill health (eg, gestational diabetes with late onset type 2 diabetes; pre-eclampsia with arterial disease). To what degree these later health conditions are a consequence of the woman's constitution prior to pregnancy rather than pregnancy itself triggering changes in a woman's health is unknown. Additionally, there is little prospective evidence for the impact of pre-pregnancy risk factors on the outcome of pregnancy. To understand the importance of pre-pregnancy health requires the recruitment of women into a long-term cohort study before their first successful pregnancy. The aim of this feasibility study is to test recruitment procedures and acceptability of participation to inform the planning of a future large-scale cohort study.

**Methods:**

The prospective cohort feasibility study will recruit nulliparous women aged 18–40 years. Women will be asked to complete a questionnaire to assess the acceptability of our recruitment and data collection procedures. Baseline biophysical, genetic, socioeconomic, behavioural and psychological assessments will be conducted and samples of blood, urine, saliva and DNA will be collected. Recruitment feasibility and retention rates will be assessed. Women who become pregnant will be recalled for pregnancy and postpregnancy assessments.

**Ethics and dissemination:**

The study protocol was approved by South Central Portsmouth REC (Ref: 12/SC/0492). The findings from the study will be disseminated through peer reviewed journals, national and international conference presentations and public events.

**Trial registration number:**

http://www.clinicaltrials.gov; NCT02419898.

**Strengths and limitations of this study**

- We recognise that there are a number of potential difficulties in the recruitment of women prior to a first successful pregnancy.
- We have designed this feasibility study to assess the practicalities of recruitment prior to pregnancy, follow-up in pregnancy and the acceptability of the study procedures.
- The results of the feasibility study will be used to inform the design of a future large cohort study.
- The feasibility study does not include long-term follow-up of the participants. This however would be an ambition of a future large cohort study.

## Introduction

The impact of maternal health on the development of the fetus and the long-term health of the child is well known.^1^ However, the impact of pregnancy on mothers’ long-term health has commanded less attention. Some specific pregnancy disorders are known to be associated with an increased incidence of long-term maternal ill health. For example, gestational diabetes with late onset type 2 diabetes,^2^ pre-eclampsia with later arterial disease^3^ and antenatal anxiety and depression with later mental health problems.^4^ It is not known to what degree these later health conditions are a consequence of the woman's constitution prior to pregnancy or whether pregnancy itself triggers permanent changes in a woman's health. For example, does a pregnancy complication such as pre-eclampsia (mainly a disease of first pregnancy^5^) simply reveal an underlying constitution susceptible to arterial disease or cause permanent changes that increase the risk of arterial disease? Currently this question has not been specifically addressed, although some studies have attempted indirectly to provide an answer.^6^ Similarly, many questions remain unanswered, for example: do antenatal anxiety and depression reveal a predisposition to mental health problems or do the hormonal and biochemical changes in pregnancy increase vulnerability to these disorders.

Pre-existing dysfunction, such as insulin resistance or hyperlipidaemia, is exacerbated by pregnancy. Hence the concept of pregnancy as a stressor for long-term chronic disease has been proposed.^7^ Problems may also be inter-related, for example insulin resistance and obesity are risk factors for both pre-eclampsia and gestational diabetes and adequate treatment of insulin resistance may reduce the risk of associated pre-eclampsia as well as ameliorate the effects of hyperglycaemia. Yet, the conditions have opposing effects on fetal growth—retarding and promoting respectively.^8^ The association between pre-pregnancy weight and risk of pre-eclampsia is well established and control of pre-pregnancy weight is considered a high priority.^9^ Linkage between population studies and birth registries has enabled the impact of hyperlipidaemia or higher blood pressures on the risk or pre-eclampsia to be estimated^6^ ^10^ but without specific knowledge of the changes during pregnancy. Whereas nutritional deficiencies of vitamin C,^11^ vitamin D,^12^ folic acid^13^ and selenium^14^ have all been linked to pre-eclampsia there is little data to show the importance of pre-pregnancy status, where intervention might be expected to have its greatest impact. Pre-eclampsia has been associated with hypothyroidism after pregnancy^15^ but the impact of pre-pregnancy thyroid status is not known. Finally excessive antiangiogenic activity is thought to be a major factor in the development of pre-eclampsia,^16^ but to what extent this reflects a long-term maternal state is completely unknown. These are some examples, related to one complication of pregnancy, but a similar dearth of knowledge applies to gestational diabetes, preterm labour and other important pregnancy problems.

The effects of socioeconomic, behavioural and psychological factors during pregnancy are well documented.^4^ ^17–20^ For example, antenatal depression affects approximately 14% of childbearing women^21^ and existing research suggests that enhanced levels of anxiety and depression symptoms during pregnancy contribute independently of other biophysical risk factors to adverse obstetric and birth outcomes.^22^ Lack of social support also constitutes an important risk factor for maternal well-being during pregnancy and has adverse effects on pregnancy outcomes.^19^ Yet the potential impact of environmental and psychological factors before pregnancy is less well understood and under-researched. Understanding the impact of these pre-pregnancy factors on the outcome of pregnancy has the potential to inform preconceptual care, identified as one of the key challenges for maternity services by National Health Service (NHS) England.^23^

The only example of a substantial pre-pregnancy cohort study is the Southampton Women's Survey (SWS).^15^ The SWS focused on fetal growth and nutrition and its impact on later health. One pilot study addressing longitudinal cardiovascular, renal and lipid function from pre-pregnancy to the postnatal period has recently been published.^24^

To distinguish the influence of pre-pregnancy from pregnancy-specific factors requires large cohort studies of women recruited before their first successful pregnancies. It is crucial to understand the effects of pre-pregnancy biophysical, genetic, socioeconomic, behavioural and psychological factors on maternal health so that women at higher risk of pregnancy and postpregnancy conditions can be identified early and supported accordingly.^24^ In addition to the effects of pregnancy on long-term maternal health, a cohort recruited prior to a first successful pregnancy affords the unique opportunity to understand the impact of pre-pregnancy maternal factors on the outcome (for the mother and baby) of the pregnancy itself. The aim of this present cohort study is therefore to characterise the health status of a community sample of young nulliparous women (aged 18–40), to determine the impact of pre-pregnancy factors on the outcome of a first pregnancy and to assess the impact of a first pregnancy, whether normal or complicated, on later maternal health. Pre-pregnancy factors that are potentially modifiable before conception will be specifically sought. The aim of this feasibility study is to test recruitment procedures and acceptability of participation in order to inform the planning of the major cohort study.

## Methods

### Study design and participants

**Figure 1:**
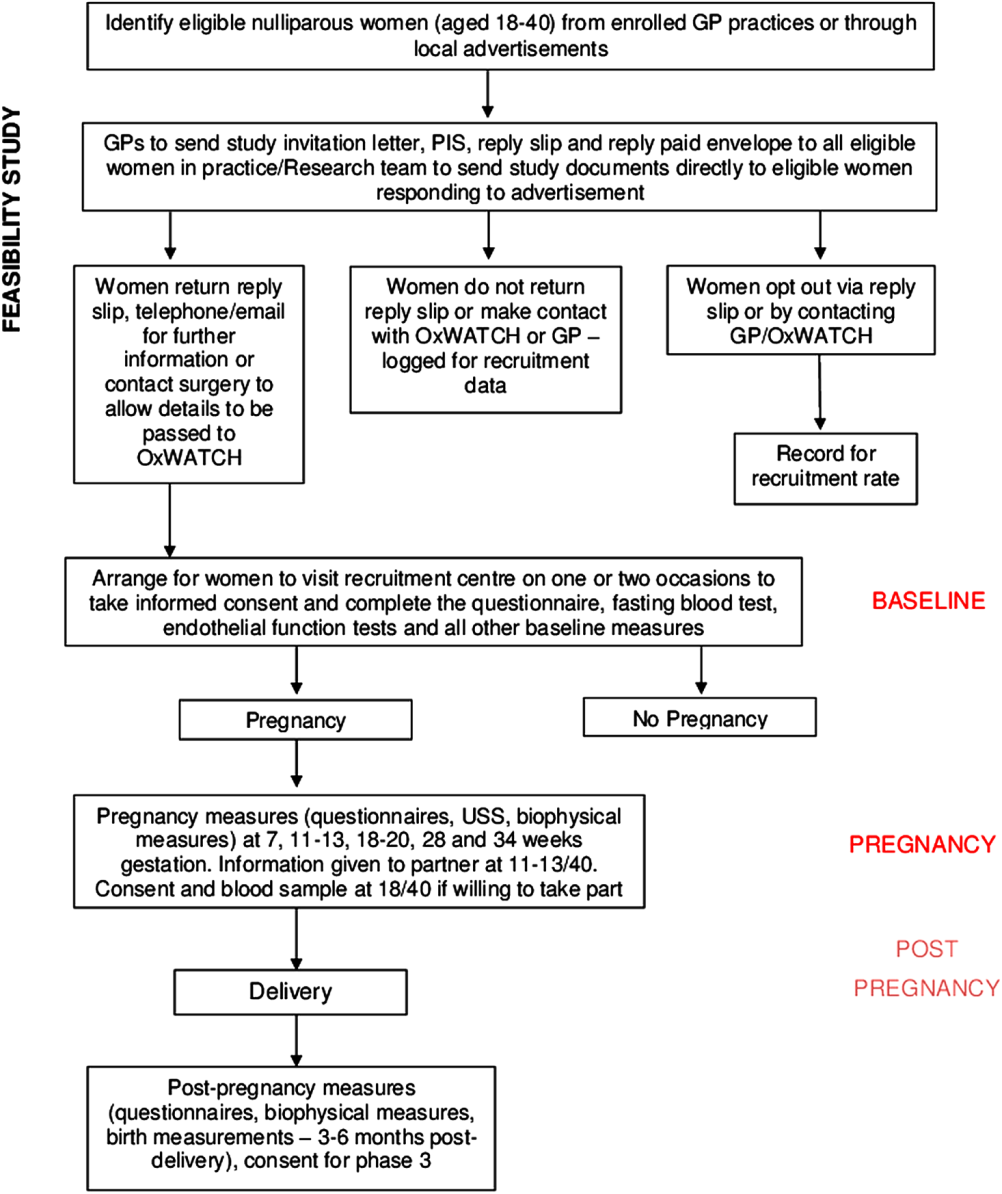
Flow chart of study recruitment.

This is a prospective cohort feasibility study. The full cohort study will have three phases (figure 1). This study will address the feasibility of phases 1 and 2 only.

- Phase 1 is a detailed characterisation of nulliparous women.
- Phase 2 is a detailed assessment of events during and immediately after a first viable pregnancy.
- Phase 3 of the cohort study is follow-up after normal and abnormal pregnancies to determine long-term pregnancy-specific sequelae for selected mothers.

Participants will be nulliparous women of reproductive age (18–40 years), who are not pregnant but could have a planned or unplanned pregnancy during the duration of the study. To meet the specific inclusion criteria participants must be: willing and able to provide informed consent; female; aged 18–40 years; not pregnant and; registered in an Oxfordshire GP practice participating in the study or have a home address in the defined geographical area. Women with a previous live birth or still birth after 24 weeks gestation are excluded from participating in the study. Potential participants will be identified from Oxfordshire GP practice lists or via local advertisement. Pre-pregnancy recruitment will take place between April 2013 and March 2016. Recruitment into phase 2 (pregnancy recruitment) will continue until March 2017 allowing 12 months for the final phase 1 recruit to become pregnant. The final postnatal visit will take place no later than March 2018.

### Study visits

Participants will attend one phase 1 baseline appointment. Once a participant has become pregnant she is eligible to enter phase 2 of the study. Pregnancy will be notified either directly or through the GP or community midwife. The vast majority of women who become pregnant in the UK will present initially to their GP, even women with secondary or tertiary care needs. Most women then receive antenatal care via NHS community services. There is very little private obstetric care in the UK. Participants will attend five visits during pregnancy at the following gestations: 7 weeks, 11–13, 18–20, 28 and 34 weeks. Finally participants will attend one postnatal visit 3 months after delivery. During the final visit, participants will be consented for future long-term follow-up (phase 3).

#### Study measures and procedures

The primary measures for the feasibility study are feasibility of recruitment and acceptability of participation. Data on recruitment pre-pregnancy, retention during and postpregnancy and overall attrition will be collected. Participants will be asked to complete a questionnaire to assess the acceptability of the recruitment and data collection procedures.

The following data will be collected: anthropometry (height, weight, body mass index, waist and hip circumferences, ultrasound measurement of abdominal and thigh fat, bioimpedence measurements); cardiovascular assessments (pulse, peripheral and central blood pressure, arterial stiffness, echocardiography, carotid imaging and endothelial function); samples of fasting and non-fasting blood, urine, saliva and DNA; questionnaire (medical, socioeconomic, behavioural and psychological assessments). The timing of study visits, study procedures and samples to be obtained are summarised in table 1. The validated questionnaires used are listed in box 1. Details of the delivery including mode of delivery and complications, gestation at delivery and neonatal outcome will also be obtained from the hospital records. The standardised assessments will be conducted by trained midwives and echocardiographers. Baseline assessments will take place at the central recruitment site or at GP practices; pregnancy and postnatal assessments will all take place at the central recruitment site.

Box 1 **Validated measures included in the questionnaire**

Spielberger Trait Anxiety Inventory

Hospital Anxiety and Depression Scale

Edinburgh Postnatal Depression Scale

EuroQol 5 Dimensions

Pain Questionnaire

Social Readjustment Rating Scale

Multidimensional Scale of Perceived Social Support

Generalized Self-Efficacy scale

**Table 1:** Data collected at each study visit

	Pre-pregnancy			Pregnancy-gestation in weeks			Postnatal
	Visit 1	Visit 2	Visit 3	Visit 4	Visit 5	Visit 6	Visit 7
	baseline	7 week	11–13 weeks	18–20 weeks	28 weeks	34 weeks	3 months
Anthropometry							
Height, weight, BMI	✓						
Weight			✓		✓	✓	✓
Hip/waist ratio	✓	✓	✓				✓
Bioimpedence	✓		✓		✓		✓
Abdominal and thigh fat ultrasound measurement	✓	✓					✓
Cardiovascular							
Pulse and BP (peripheral and central) and arterial stiffness	✓	✓	✓		✓	✓	✓
Echocardiograph, carotid imaging, and endothelial function	✓		✓		✓		✓
Blood							
Fasting	✓		✓		✓		✓
Non-fasting		✓		✓		✓	
DNA	✓ (maternal)			✓ (paternal)			
Saliva	✓	✓	✓	✓	✓		
Urine	✓	✓	✓	✓	✓	✓	✓
Fetal ultrasound		✓	✓	✓	✓		
Questionnaire							
Socioeconomic information	✓	✓			✓		✓
Medical history	✓	✓					
Pregnancy history	✓	✓					
Family history	✓	✓			✓		✓
Tobacco/alcohol/drug use	✓	✓			✓		✓
Diet and exercise	✓	✓			✓		✓
Mental health and well-being study acceptability	✓	✓			✓		✓

BMI, body mass index; BP, blood pressure.

### Outcomes

#### Primary outcome

The recruitment of 300 nulliparous women into phase 1 and the follow-up of 100 women into phase 2 to prove feasibility of the cohort study.

#### Secondary outcomes

##### Recruitment and data collection

1. The retention of participants in phase 2
2. The incidence of pregnancy within 12 months of recruitment
3. The live birth rate
4. The incidence of pregnancy loss in phase 2 participants
5. The incidence of loss to follow-up
6. The proportion of participants who provide full data at baseline and throughout pregnancy

##### Acceptability

1. The acceptability of the recruitment strategy to participants
2. The acceptability of the data collection procedures to participants

### Sample size

As this is a feasibility study to test recruitment and acceptability, no formal power calculations were undertaken. Eight four per cent of women planning a pregnancy will conceive within 12 months and 25% of nulliparous 20–35 year olds will deliver a child within 3 years.^25^ Data from the SWS (where nulliparous women were recruited regardless of pregnancy intention) showed that almost 25% of pregnancies within 3 months of recruitment were unplanned. Although targeting only women planning pregnancy would yield the highest pregnancy rate, that approach may heavily bias the type of individual recruited. We aim to recruit approximately 150 women planning a pregnancy and a similar number regardless of pregnancy intention. We estimate this will result in 160 pregnancies in the time frame of the study. Given that at least 20% of pregnancies will result in miscarriage^26^ this is likely to result in approximately 130 live births. This number of pregnancies will allow us to adequately test pregnancy recruitment and retention rates, study procedures and their acceptability to recruits.^27^

### Data analysis plan

Analysis of the feasibility study will be descriptive.^28^ The number of participants recruited into phases 1 and 2 during the feasibility study recruitment period will be reported. This will enable us to calculate the staffing requirements, time plan, and the number of centres required to ensure we will recruit to target and on time in the cohort study.

Numbers of pregnancies, live births, adverse events, full data sets and losses to follow-up will be reported as percentages of the total number of participants/pregnancies. This data will enable us to calculate attrition rates for the cohort study. Together with established incidence rates for the outcomes of interest in the cohort study, this will allow us to assess incidence locally and to calculate the number of participants we require at baseline.

Preliminary analysis of the planned cohort study outcomes will be conducted to validate the analysis plan. In addition, the investigators anticipate reporting the longitudinal data obtained from the pilot study.

## Ethics and dissemination

The OxWATCH feasibility study is conducted in accordance with the ‘Helsinki Declaration’ (1996). The study is observational and non-invasive, imposes no risk on participants, and its protocol has been approved by the South Central Portsmouth Research Ethics Committee (Ref: 12/SC/0492) and by the Research and Development departments of all participating NHS trusts. Furthermore, written informed consent is obtained from all participants.

If any of the fetal ultrasound scans or other study assessments suggest a clinical problem the patient will be referred, with their consent, to the relevant specialist at their local hospital. This is explained to participants before joining the study.

The findings from the feasibility study will be disseminated to the medical, scientific and public through peer reviewed journals, national and international conference presentations, and public events. The feasibility study findings will also be used to plan and improve the cohort study.

Trial status: phase 1 feasibility study recruitment is planned to complete in March 2016. Phase 2 study recruitment is planned to complete in March 2017. Data collection will end once the final pregnant recruit has attended a postnatal assessment.
